# Nipple Reconstruction Using the C-V Flap Technique: Long-Term Outcomes and Patient Satisfaction

**Published:** 2017-01

**Authors:** Lona Jalini, Jonathan Lund, Vijay Kurup

**Affiliations:** 1 North Tees and Hartlepool University Hospital NHS Foundation Trust, United Kingdom;; 2 Breast and Endocrine Surgeon, North Tees and Hartlepool University Hospital NHS Foundation Trust, United Kingdom

**Keywords:** C-V flap, Nipple reconstruction, Patient satisfaction

## Abstract

**BACKGROUND:**

Nipple creation using the C-V flap technique is often the final step in breast reconstruction. The aim of this study was to subjectively and objectively assess the cosmetic outcomes and satisfaction of patients undergoing C-V flap nipple reconstruction.

**METHODS:**

Subjective assessments of patient satisfaction with the neo-nipple were recorded by visual analogue scoring (VAS; 0-10). Objective measurements were performed using a calliper to measure nipple projection relative to the native breast. Descriptive data analysis was performed with differences in projection assessed with the Mann-Whitney test and mean and median VAS scores (with inter-quartile ranges; IQR) calculated to describe satisfaction.

**RESULTS:**

Thirty-three C-V flap nipple reconstructions were performed. 87.9% received latissimus dorsi (LD) reconstructions with implants and 12.1% had transverse rectus abdominis muscle (TRAM) reconstructions. The median projection of reconstructed nipples was 4.7 mm (range 4-10.2 mm) at 4.6 years mean follow-up, which was not significantly different from the contralateral nipple (p = 0.34). Patient satisfaction was 9 (IQR: 8-10) with shape, 9 (IQR: 7.5-10) with projection, 5 (IQR: 2-9.6) with sensation, and 8.5 (IQR: 6-9.5) with symmetry. Median overall satisfaction was 9 (IQR: 8-10). Three patients had complete nipple loss, of whom two had undergone nipple piercing post procedure and none had received radiotherapy.

**CONCLUSION:**

C-V flap nipple reconstructions provide a simple and reliable method to reconstruct the nipple that enhances confidence and perception of body image. Satisfaction was high with long-term outcomes in terms of projection equivalent to the contralateral breast.

## INTRODUCTION

Nipple reconstruction is the final phase in the long journey of breast reconstruction. When performed well, the patient can finally experience and view the reconstructed breast as normal.^1^ Nipple reconstruction is often combined with tattooing to simulate not only areolar colour but also to camouflage the scar. However, tattooing is not always necessary, and some women decide to undergo nipple reconstruction without tattooing and vice versa. One challenge in nipple reconstruction is to produce a three-dimensional structure from a two-dimensional surface.^1^

Two basic methods are used to achieve this: first, reconstruction using the local flap with or without tattooing or skin grafting from various donor sites (e.g., the inner thigh or buttock crease), and second, the free nipple graft, which is a composite flap using cartilage from another part of the body,^2^ filler material (e.g., AlloDerm, Radiesse),^3^ or tissue from the contralateral breast (the so-called ‘nipple sharing’ technique). All these techniques suffer from some loss of projection over time as part of the normal healing process and formation of scar contracture.^3^ The C-V flap was first described in 1998^4^ and offers a simple but reliable nipple reconstruction method that can easily be learned and performed under local or general anaesthesia with or without tattooing eight to twelve weeks after cancer surgery. 

The procedure is associated with good satisfaction rates and a positive impact on body image and confidence.^5^ Complications include bruising, infection, delayed wound healing, and, most seriously but rarely, complete or partial nipple loss. The aim of this study was to objectively and subjectively assess the cosmetic outcomes and satisfaction of patients undergoing C-V flap nipple reconstruction under the care of a single oncoplastic breast surgeon at a screening unit for service modification and improvement.

## MATERIALS AND METHODS

All patients undergoing C-V flap nipple reconstruction under the care of V.K. between 2006 and 2015 were identified from the hospital breast cancer database. There were no exclusion criteria. Case notes were retrieved and demographic information including age, date of surgery, past medical history, smoking history, type of breast reconstruction, symmetrising procedure, pre- or post-operative radiotherapy, and complications were documented. 

Subjective assessments were made using a questionnaire focusing primarily on patient satisfaction. Visual analogue scoring (VAS) from 0 to 10 (with 0 as the worst and 10 as the best possible outcome) was used to record patient satisfaction with projection, sensation, symmetry, and willingness to recommend the procedure to other women. Objective measurements were made using a calliper to measure the nipple projection in relation to the native breast. The Mann-Whitney U-test was used to test for differences in projection between the reconstructed nipple and contralateral nipple; a p value ≤ 0.05 was regarded as significant. 

VAS scores were analysed to determine the mean and median satisfaction with inter-quartile ranges (IQR). Ethical approval was not required for this study since this was a review of service provision, but all patients verbally consented to participate. All patients were marked sitting upright. The position of the C-V flap was marked in relation to the nipple position in the native breast and whether a symmetrising procedure was planned on the contralateral side. The flap was marked to ensure that the blood supply was away from any old scar. The flap was designed one and a half to twice the size of the contralateral nipple to allow for 50% shrinkage or reduction in projection occurring over time due to absorption of the central fat core to optimise the long-term cosmesis. 

**Fig. 1:**
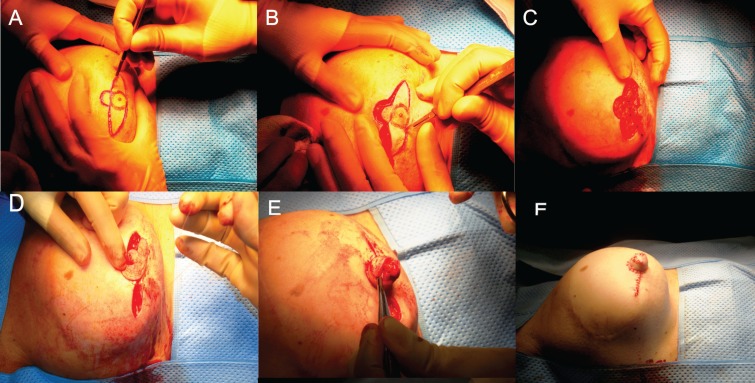
The operative technique

The procedure was carried out under general anaesthesia. The design and incision of the flap is shown in Figures (1 A, B, C). Flaps were composed of two lateral V flaps and a central C-shaped flap: the diameter of the central C-shaped flap determined the diameter of the new nipple, while the projection was determined by the width of the V flaps. The base of the C-shaped dermal flap remained attached since the blood supply is derived from the sub-dermal plexus from the un-incised portion of skin. The V flaps were elevated from the underlying subcutaneous tissue and wrapped around and sutured to the central C flap with 4-0 Monocryl (Ethicon, Johnson and Johnson, New Brunswick, NJ). 

The V flap donor sites were then closed with non-absorbable 4-0 nylon interrupted sutures (Figures 1 D, E, F). Some authors^1^ suggest using a protective shield filled with antibiotic ointment or an eye bubble for two weeks post-operatively, whilst others use Lyofoam circles for a further four weeks to protect the new nipple.^6^ We chose to apply waterproof, non-crushing adhesive dressing with a window to allow inspection of the viability of the reconstructed nipple. The dressing was left undisturbed for two weeks for post-operative protection.

## RESULTS

Thirty-two women underwent 33 C-V flap nipple reconstructions in our unit between 2006 and 2015. All patients attended clinic for measurements to compare nipple projection in both breasts. Patient demographics are shown in Table 1. The mean age at reconstruction was 53.5 years (median 52 years, range 40-67 years). Mean and median follow-up were 4.6 and 4 years, respectively (range 3-108 months). No patient was a smoker or diabetic at the time of surgery. Twenty-nine patients (87.9%) had latissimus dorsi (LD) reconstructions with implants and four (12.1%) had transverse rectus abdominis muscle (TRAM) reconstructions. Seven (21.2%) had radiotherapy before nipple reconstruction. A total of nine patients (27.3%) had contralateral breast reduction or mastopexy 12-18 months after nipple reconstruction. Twenty-one (63.6%) patients were tattooed post-operatively. 

**Table 1:** Patient demographics, operative details, and outcomes

**Variable**	**n (%), unless otherwise stated**
Total no. C-V flaps	33
Age (range, mean, median; yrs)	40 – 67, 53.5, 52
Follow-up (range (m), mean, median (yrs)	3 – 108, 4.6, 4
Pre-operative radiotherapy	7 (21)
Current smoker	0 (0)
Diabetic	0 (0)
Type of reconstruction:	
Latissimus dorsi + implant	29 (87.9)
TRAM	4 (12.1)
Symmetrisation	9 (27.3)
Tattooing	21 (63.6)
Flap loss	3 (9.1)

The median projection of the C-V flap was 4.7 mm (range 4-10.2 mm) with thirteen patients having nipple projection of greater than 4.2 mm at 4.6 years mean follow-up. The overall difference in projection between flap and contralateral side was not statistically significant (*p*=0.34). Patients reported a mean VAS of 8.3 and median of 9 (IQR: 8-10) with shape, a mean of 8.1 and median of 9 (IQR: 7.5-10) with projection, a mean of 5.4 and median of 5 (IQR: 2-9.5) with sensation, and a mean of 7.3 and median of 8.5 (IQR: 6-9.5) with symmetry. Mean overall satisfaction was 8.2 with a median of 9 (IQR: 8-10). 

Eighty-nine per cent of women were happy to recommend the procedure to other women (Table 2). Twenty-one women were completely satisfied with all aspects of their care including surgery, while the remainder (eleven in total) raised various concerns (Table 3). In terms of clinical outcomes, partial ischemic necrosis occurred in seven (21.2%) patients, of whom three experienced complete nipple loss. None had received radiotherapy, but two women with complete nipple loss underwent nipple piercing against medical advice.

**Table 2:** Patient satisfaction with C-V nipple reconstruction scored using a visual analogue scale (IQR)

**Parameter**	**Mean score**	**Median score**
Shape	8.3	9 (8-10)
Projection	8.1	9 (7.5-10)
Sensation	5.4	5 (2-9.5)
Symmetry	7.3	8.5 (6-9.5)
Overall satisfaction	8.2	9 (8-10)
Confidence post-procedure	8.4	10 (8-10)
Recommend to other women	8.9	10 (8.8-10)

**Table 3:** Specific concerns raised by patients with respect to outcome

**Variable**	**N**	**%**
Completely satisfied	21	63.6
Intractable itch	1	3.0
Tattoo fading	1	3.0
Greater projection desired	4	12.1
Loss of nipple (complete)	3	9.1
Improved waiting time	1	3.0

## DISCUSSION

Although a number of nipple reconstruction procedures are described in the literature, few clinical trials have been conducted to reach a consensus on a favoured method in terms of long-term cosmesis and ease of the technique. In practice, the chosen method is usually dependent on the experience of the individual surgeon and patient choice. Nipple reconstruction with or without areola tattooing is the finishing touch and the defining feature of the female breast.^7^ Some studies have shown that timely reconstruction leads to improved psychological wellbeing in the patient and improved patient and partner satisfaction.^5^^,^^8^

Regardless of the technique employed, certain rules are followed to achieve a successful local flap reconstruction including leaving a wide enough pedicle to ensure adequate blood supply while detaching it from surrounding tissue to allow flap shaping. Most reconstructed nipples retract over time due to scarring and scar contraction, particularly when there has been previous radiotherapy, infection, or poor flap design that compromises the circulation and delays healing. Well established flaps include the skate,^9^ star,^10^ double-opposing tab flap,^11^^-^^13^ double opposing V-Y flap,^14^ and the V-Y advancement flap.^15^

Since its introduction in 1998, the C-V flap has been shown to be a successful method, albeit with a variable rate of patient satisfaction ranging from low to high.^4^^,^^16^ The most common dissatisfaction with nipple reconstruction is flattening and loss of projection over time followed by colour mismatch, shape, size, and malposition.^17^ Here we report a high satisfaction rate with shape (median VAS 9) and symmetry (median VAS 8.5), with the majority of patients feeling more confident about their body image. Only one patient reported tattoo fading, but this was three years post procedure. 

Another patient reported intractable itching eighteen months post procedure. Patients were informed at the time of surgery that nipple reconstruction cannot fully restore normal sensation, reflected in a median overall satisfaction of five for sensation. Losken *et al.*^4^ analysed the long-term projection of C-V flaps in fourteen cases after an average follow up of 5.3 years. The average projection was 3.77 mm with a patient satisfaction of 42%. Similarly, Jabor *et al.*^5^ showed that, in most cases, the excessive loss of projection over time is the principal cause of dissatisfaction in over 50 percent of women. 

Eo *et al.*^18^ reported improved projection with the use of excess tissue at mound revision. More recently, Park *et al.*^6^ reported satisfactory outcomes in 18 cases by designing a composite C-V flap in which a free dermal flap was used from various donor sites (the lateral dog-ears from LD, TRAM, or deep inferior epigastric perforator (DIEP) scars) to augment the C-V flap. There was a high satisfaction of 73% with the cosmetic appearance after an average follow-up of 36.8 months in the current study.

In a large study of 252 C-V flap nipple reconstructions,^19^ the overall complication rate was reported as 4% (0.8% wound infections and 3.2% tip necrosis), with 64% patient satisfaction. Thirty-eight per cent of patients, however, wanted greater projection. In our study, three patients suffered complete flap loss (9.1%) but two had undergone nipple piercing post operatively against medical advice. Those with complete nipple loss scored the lowest overall satisfaction but would still have recommended the procedure to other women. There was no association between partial nipple necrosis or nipple loss and previous radiotherapy. 

No significant difference in projection was noted between the reconstructed nipple and the native nipple. Our study, however, included three patients six to twelve weeks post-operative in whom no loss of projection had yet occurred. Our results are in agreement with other publications reporting high satisfaction rates between 62 and 81%.^6^^,^^19^^-^^21^ We speculate that the presence of implants under LD flaps might have exerted an additional upward force on the skin surface to maintain better projection. Several studies have shown that, after surgery, the bandage should not compress the reconstructed nipple since this can contribute to its flattening in the long term.^4^^,^^13^^,^^22^ Loss of projection is a common problem over time.^17^ To address this, there have been attempts to augment the C-V flap using acellular dermal matrix,^1^^,^^2^ conchal cartilage,^23^ or silicon rods^24^ with variable complication and success rates. However, the search for an ideal method continues.

In conclusion, the C-V flap provides a simple and reliable method of nipple reconstruction that enhances confidence and perception of body image. The satisfaction rate at our unit was high and the long-term outcome of projection was not significantly different from the contralateral breast. This reflected a conscious decision by the operating surgeon to design a larger flap to accommodate later shrinkage. Our study was limited by a relatively small sample size from a single institution and there was potential for observation bias when measuring nipple projection. 

Despite being a retrospective study, the follow-up period was substantial and prospective data collection is currently being carried out to further assess long-term outcomes in all patients undergoing this procedure in our unit. The introduction of new techniques to address loss of projection in some patients provides new opportunities to improve appearance of the “finishing touch” and focal point of the reconstructed female breast.

## CONFLICT OF INTEREST

The authors declare no conflict of interest.

## References

[1] Nahabedian MY (2007). Nipple reconstruction. Clin Plast Surg.

[2] Yanaga H (2003). Nipple-areola reconstruction with a dermal-fat flap: technical improvement from rolled auricular cartilage to artificial bone. Plast Reconstr Surg.

[3] Nahabedian MY (2005). Secondary nipple reconstruction using local flaps and AlloDerm. Plast Reconstr Surg.

[4] Losken A, Mackay GJ, Bostwick J 3rd (2001). Nipple reconstruction using the C-V flap technique: a long-term evaluation. Plast Reconstr Surg.

[5] Jabor MA, Shayani P, Collins DR Jr, Karas T, Cohen BE (2002). Nipple-areola reconstruction: satisfaction and clinical determinants. Plast Reconstr Surg.

[6] Elizabeth Clark S, Turton E (2014). The CC-V Flap: A Novel Technique for Augmenting a C-V Nipple Reconstruction Using a Free Dermal Graft. World J Plast Surg.

[7] Few JW, Marcus JR, Casas LA, Aitken ME, Redding J (1999). Long-term predictable nipple projection following reconstruction. Plast Reconstr Surg.

[8] Wellisch DK, Schain WS, Noone RB, Little JW, 3rd (1987). The psychological contribution of nipple addition in breast reconstruction. Plast Reconstr Surg.

[9] Zhong T, Antony A, Cordeiro P (2009). Surgical outcomes and nipple projection using the modified skate flap for nipple-areolar reconstruction in a series of 422 implant reconstructions. Ann Plast Surg.

[10] Gurunluoglu R, Shafighi M, Williams SA, Kimm GE (2012). Incorporation of a preexisting scar in the star-flap technique for nipple reconstruction. Ann Plast Surg.

[11] Kroll SS (1999). Nipple reconstruction with the double-opposing tab flap. Plast Reconstr Surg.

[12] Kroll SS, Hamilton S (1989). Nipple reconstruction with the double-opposing-tab flap. Plast Reconstr Surg.

[13] Kroll SS, Reece GP, Miller MJ, Evans GR, Robb GL, Baldwin BJ, Wang BG, Schusterman MA (1997). Comparison of nipple projection with the modified double-opposing tab and star flaps. Plast Reconstr Surg.

[14] Lesavoy M, Liu TS (2010). The diamond double-opposing V-Y flap: a reliable, simple, and versatile technique for nipple reconstruction. Plast Reconstr Surg.

[15] Riccio CA, Zeiderman MR, Chowdhry S, Wilhelmi BJ (2015). Review of nipple reconstruction techniques and introduction of v to y technique in a bilateral wise pattern mastectomy or reduction mammaplasty. Eplasty.

[16] El-Ali K, Dalal M, Kat CC (2009). Modified C-V flap for nipple reconstruction: our results in 50 patients. J Plast Reconstr Aesthet Surg.

[17] Hammond DC, Khuthaila D, Kim J (2007). The skate flap purse-string technique for nipple-areola complex reconstruction. Plast Reconstr Surg.

[18] Eo S, Kim SS, Da Lio AL (2007). Nipple reconstruction with C-v flap using dermofat graft. Ann Plast Surg.

[19] Otterburn DM, Sikora KE, Losken A (2010). An outcome evaluation following postmastectomy nipple reconstruction using the C-V flap technique. Ann Plast Surg.

[20] Momoh AO, Colakoglu S, de Blacam C, Yueh JH, Lin SJ, Tobias AM, Lee BT (2012). The impact of nipple reconstruction on patient satisfaction in breast reconstruction. Ann Plast Surg.

[21] Valdatta L, Montemurro P, Tamborini F, Fidanza C, Gottardi A, Scamoni S (2009). Our experience of nipple reconstruction using the C-V flap technique: 1 year evaluation. J Plast Reconstr Aesthet Surg.

[22] Weinfeld AB, Somia N, Codner MA (2008). Purse-string nipple areolar reconstruction. Ann Plast Surg.

[23] Jones AP, Erdmann M (2012). Projection and patient satisfaction using the “Hamburger” nipple reconstruction technique. J Plast Reconstr Aesthet Surg.

[24] Jankau J, Jaskiewicz J, Ankiewicz A (2011). A new method for using a silicone rod for permanent nipple projection after breast reconstruction procedures. Breast.

